# Crystal structure of dimethyl 3,3′-[(4-fluoro­phen­yl)methyl­ene]bis­(1*H*-indole-2-carboxyl­ate)

**DOI:** 10.1107/S205698901501628X

**Published:** 2015-09-12

**Authors:** Hong-Shun Sun, Yu-long Li, Hong Jiang, Ning Xu, Hong Xu

**Affiliations:** a Chemical Engineering Department, Nanjing Polytechnic Institute, Nanjing 210048, People’s Republic of China

**Keywords:** crystal structure, MRI contrast agent, indole, bis-indolymethane, N—H⋯O hydrogen bonds, C—H⋯π inter­actions

## Abstract

In the title compound, C_27_H_21_FN_2_O_4_, the mean planes of the indole ring systems (r.m.s. deviations = 0.0263 and 0.0160 Å) are approximately perpendic­ular to one another, making a dihedral angle of 84.0 (5)°; the fluoro­benzene ring is twisted with respect to the mean planes of the two indole ring systems at 89.5 (5) and 84.6 (3)°. In the crystal, pairs of N—H⋯O hydrogen bonds link the mol­ecules into inversion dimers, which are further linked by N—H⋯O hydrogen bonds into supra­molecular chains propagated along the *b*-axis direction. Weak C—H⋯π inter­actions are observed between neighbouring chains.

## Abstract 2

In the title compound, the two indole ring systems are approximately perpendicular to one another, with a dihedral angle between their planes of 84.0 (5)°.

## Chemical context   

Bis(indol­yl)methane derivatives have been found widely in various terrestrial and marine natural resources (Porter *et al.*, 1977 ▸; Sundberg, 1996 ▸), and have many applications in pharmaceuticals with diverse activities, such as anti­cancer, anti­leishmanial and anti­hyperlipidemic (Chang *et al.*, 1999 ▸; Ge *et al.*, 1999 ▸). Other, bis­(indoly)methane derivatives can also be used as a precursor for MRI contrast agents (Ni, 2008 ▸). In recent years, we have reported the synthesis and crystal structures of some similar compounds (Sun *et al.*, 2012 ▸, 2013 ▸, 2014 ▸; Li *et al.*, 2014 ▸; Lu *et al.*, 2014 ▸). Now we report herein another bis­(indoly)methane compound.
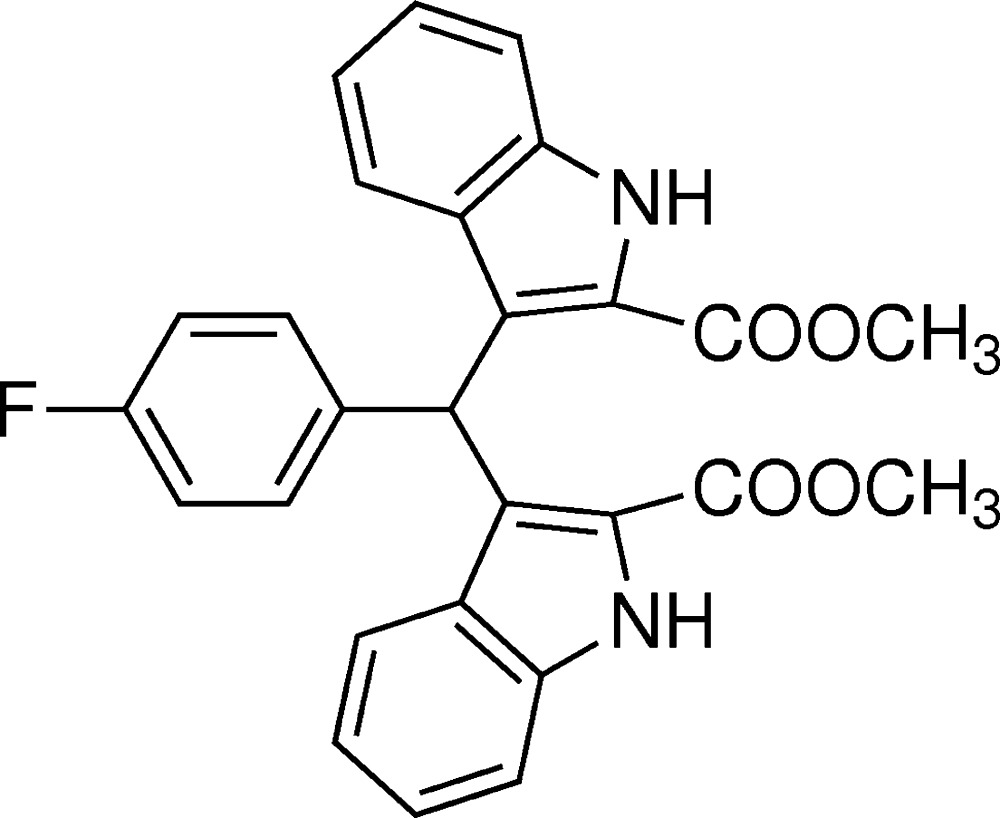


## Structural commentary   

**Figure 1:**
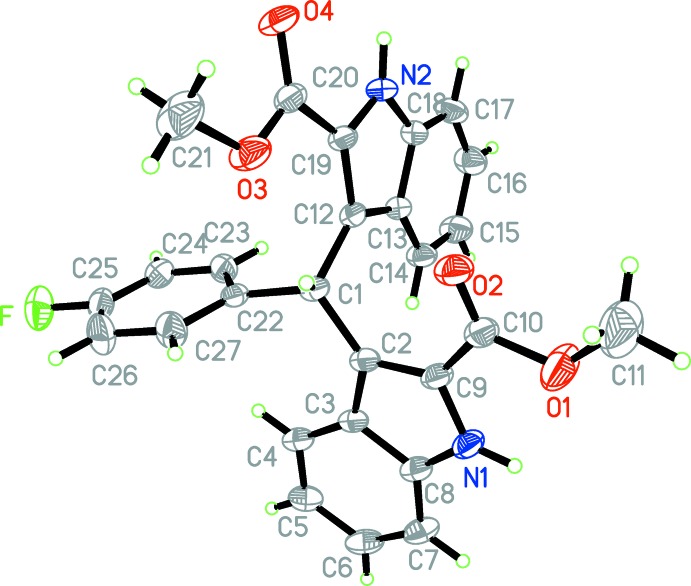
The mol­ecular structure of the title mol­ecule, showing the atom-labelling scheme. Displacement ellipsoids are drawn at the 30% probability level.

The mol­ecular structure of the title compound is shown in Fig. 1 ▸. The two indole ring systems are approximately planar, the maximum deviations being 0.049 (3) and 0.030 (2) Å; the mean planes of the two indole ring systems nearly perpen­dicular to each other [dihedral angle = 84.0 (5)°] while the benzene ring (C22–C27) is twisted to the N1/C2–C9 and N2/C12–C19 indole ring systems by dihedral angles of 89.5 (5) and 84.6 (3)°, respectively. The carboxyl groups are approximately co-planar with the attached indole ring systems, the dihedral angles between the carboxyl groups and the mean planes of attached indole ring systems are 10.8 (3) and 12.3 (4)°.

## Supra­molecular features   

**Figure 2:**
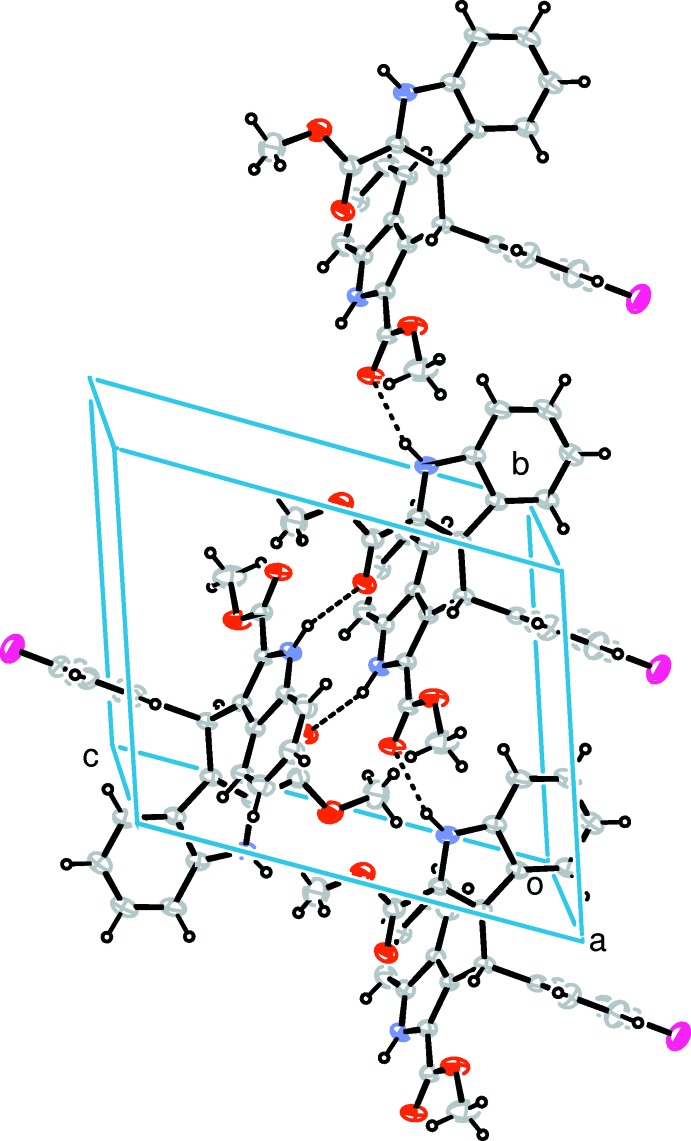
A packing diagram of the title compound. Hydrogen bonds are shown as dashed lines.

In the crystal, pairs of N1—H1*A*⋯O4^i^ [symmetry code: (i) *x*, *y* + 1, *z*] hydrogen bonds link the mol­ecules into inversion dimmers, which are further linked by N2—H2*A*⋯O2^ii^ [symmetry code: (ii) 1 − *x*, 1 − *y*, 1 − *z*] hydrogen bonds into supra­molecular chains propagated along the *b* axis (Table 1 ▸ and Fig. 2 ▸). Weak C—H⋯π inter­actions are also observed between neighbouring chains.

## Database survey   

Several similar structures have been reported previously, *i.e.* diethyl 3,3′-(phenyl­methyl­ene)bis­(1*H*-indole-2-carboxyl­ate) (Sun *et al.*, 2012 ▸), dimethyl 3,3′-(phenyl­methyl­ene)bis­(1*H*-indole-2-carboxyl­ate) (Sun *et al.*, 2013 ▸), dimethyl 3,3′-[(4-chloro­phen­yl) methyl­ene]bis­(1*H*-indole-2-carboxyl­ate) (Li *et al.*, 2014 ▸), dimethyl 3,3′-[(3-nitro­phen­yl) methyl­ene]bis­(1*H*-indole-2-carboxyl­ate) ethanol monosolvate (Sun *et al.*, 2014 ▸) and dimethyl 3,3′-[(3-fluoro­phen­yl)methyl­ene]bis­(1*H*-indole-2-carboxyl­ate) (Lu *et al.*, 2014 ▸). In those structures, the two indole ring systems are also nearly perpendicular to each other, the dihedral angles being 82.0 (5), 84.5 (5), 79.5 (4), 89.3 (5) and 87.8 (5)°, respectively.

## Synthesis and crystallization   

Methyl indole-2-carboxyl­ate (17.5 g, 100 mmol) was dissolved in 200 ml methanol; commercially available 4-fluoro­benzaldehyde (6.2 g, 50 mmol) was added and the mixture was heated to reflux temperature. Concentrated HCl (3.7 ml) was added and the reaction was left for 1 h. After cooling the white product was filtered off and washed thoroughly with methanol. The reaction was monitored by TLC (CHCl_3_:hexane = 1:1). Yield was 90%. Single crystals of the title compound suitable for X-ray analysis were obtained by slow evaporation of a methanol solution. ^1^H NMR (300 MHz, DMSO) δ 11.81 (*s*, 2H), 7.59–7.36 (*m*, 3H), 7.13 (*dd*, *J* = 15.1, 7.2 Hz, 6H), 6.71 (*t*, *J* = 7.5 Hz, 2H), 6.60 (*d*, *J* = 8.3 Hz, 2H), 3.77 (*s*, 6H).

## Refinement   

Crystal data, data collection and structure refinement details are summarized in Table 2 ▸. H atoms were positioned geometrically with N—H = 0.86 Å and C—H = 0.93–0.98 Å, and constrained to ride on their parent atoms. *U*_iso_(H) = *xU*_eq_(C,N), where *x* = 1.5 for methyl H atoms and 1.2 for the others.

## Supplementary Material

Crystal structure: contains datablock(s) I, global. DOI: 10.1107/S205698901501628X/xu5870sup1.cif

Structure factors: contains datablock(s) I. DOI: 10.1107/S205698901501628X/xu5870Isup2.hkl

Supporting information file. DOI: 10.1107/S205698901501628X/xu5870Isup3.cml

CCDC reference: 1421585

Additional supporting information:  crystallographic information; 3D view; checkCIF report

## Figures and Tables

**Table 1:** Hydrogen-bond geometry (, ) *Cg*1, *Cg*4 and *Cg*5 are the centroids of the N1-ring, C14-ring and C22-ring.

*D*H*A*	*D*H	H*A*	*D* *A*	*D*H*A*
N1H1*A*O4^i^	0.86	2.07	2.903(3)	162
N2H2*A*O2^ii^	0.86	2.07	2.892(3)	159
C4H4*A* *Cg*5	0.93	2.82	3.622(3)	146
C5H5*B* *Cg*4^iii^	0.93	2.84	3.705(3)	156
C26H26*A* *Cg*1^iv^	0.93	2.69	3.587(4)	161

Symmetry codes: (i) 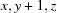; (ii) 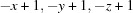; (iii) 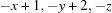; (iv) 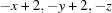.

**Table 2:** Experimental details

Crystal data	
Chemical formula	C_27_H_21_FN_2_O_4_
*M*_r_	456.46
Crystal system, space group	Triclinic, *P* 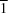
Temperature (K)	293
*a*, *b*, *c* ()	9.7270(19), 10.122(2), 13.441(3)
, , ()	68.15(3), 73.69(3), 89.73(3)
*V* (^3^)	1171.4(4)
*Z*	2
Radiation type	Mo *K*
(mm^1^)	0.09
Crystal size (mm)	0.30 0.20 0.10

Data collection	
Diffractometer	Nonius CAD-4 diffractometer
Absorption correction	scan (North *et al.*, 1968 ▸)
*T*_min_, *T*_max_	0.973, 0.991
No. of measured, independent and observed [*I* > 2(*I*)] reflections	4587, 4311, 2756
*R*_int_	0.040
(sin /)_max_ (^1^)	0.603

Refinement	
*R*[*F* ^2^ > 2(*F* ^2^)], *wR*(*F* ^2^), *S*	0.054, 0.147, 1.00
No. of reflections	4311
No. of parameters	307
H-atom treatment	H-atom parameters constrained
_max_, _min_ (e ^3^)	0.16, 0.18

Computer programs: *CAD-4 EXPRESS* (EnrafNonius, 1994 ▸), *XCAD4* (Harms Wocadlo, 1995 ▸), *SHELXTL* (Sheldrick, 2008 ▸).

## supplementary crystallographic information

### Crystal data

**Table:**

C_27_H_21_FN_2_O_4_	*Z* = 2
*M**_r_* = 456.46	*F*(000) = 476
Triclinic, *P*1	*D*_x_ = 1.294 Mg m^−^^3^
Hall symbol: -P 1	Mo *K*α radiation, λ = 0.71073 Å
*a* = 9.7270 (19) Å	Cell parameters from 25 reflections
*b* = 10.122 (2) Å	θ = 9–13°
*c* = 13.441 (3) Å	µ = 0.09 mm^−^^1^
α = 68.15 (3)°	*T* = 293 K
β = 73.69 (3)°	Block, colorless
γ = 89.73 (3)°	0.30 × 0.20 × 0.10 mm
*V* = 1171.4 (4) Å^3^	

### Data collection

**Table:**

Nonius CAD-4 diffractometer	2756 reflections with *I* > 2σ(*I*)
Radiation source: fine-focus sealed tube	*R*_int_ = 0.040
Graphite monochromator	θ_max_ = 25.4°, θ_min_ = 1.7°
ω/2θ scans	*h* = 0→11
Absorption correction: ψ scan (North *et al.*, 1968)	*k* = −12→12
*T*_min_ = 0.973, *T*_max_ = 0.991	*l* = −15→16
4587 measured reflections	3 standard reflections every 200 reflections
4311 independent reflections	intensity decay: 1%

### Refinement

**Table:**

Refinement on *F*^2^	Primary atom site location: structure-invariant direct methods
Least-squares matrix: full	Secondary atom site location: difference Fourier map
*R*[*F*^2^ > 2σ(*F*^2^)] = 0.054	Hydrogen site location: inferred from neighbouring sites
*wR*(*F*^2^) = 0.147	H-atom parameters constrained
*S* = 1.00	*w* = 1/[σ^2^(*F*_o_^2^) + (0.075*P*)^2^] where *P* = (*F*_o_^2^ + 2*F*_c_^2^)/3
4311 reflections	(Δ/σ)_max_ < 0.001
307 parameters	Δρ_max_ = 0.16 e Å^−^^3^
0 restraints	Δρ_min_ = −0.18 e Å^−^^3^

### Special details

**Table:**

Geometry. All e.s.d.'s (except the e.s.d. in the dihedral angle between two l.s. planes) are estimated using the full covariance matrix. The cell e.s.d.'s are taken into account individually in the estimation of e.s.d.'s in distances, angles and torsion angles; correlations between e.s.d.'s in cell parameters are only used when they are defined by crystal symmetry. An approximate (isotropic) treatment of cell e.s.d.'s is used for estimating e.s.d.'s involving l.s. planes.
Refinement. Refinement of *F*^2^ against ALL reflections. The weighted *R*-factor *wR* and goodness of fit *S* are based on *F*^2^, conventional *R*-factors *R* are based on *F*, with *F* set to zero for negative *F*^2^. The threshold expression of *F*^2^ > σ(*F*^2^) is used only for calculating *R*-factors(gt) *etc*. and is not relevant to the choice of reflections for refinement. *R*-factors based on *F*^2^ are statistically about twice as large as those based on *F*, and *R*- factors based on ALL data will be even larger.

### Fractional atomic coordinates and isotropic or equivalent isotropic displacement parameters (Å^2^)

**Table:**

	*x*	*y*	*z*	*U*_iso_*/*U*_eq_	
N1	0.6800 (2)	1.1318 (2)	0.27546 (18)	0.0513 (6)	
H1A	0.6727	1.1742	0.3213	0.062*	
C1	0.7071 (2)	0.8060 (2)	0.20833 (19)	0.0382 (5)	
H1B	0.7844	0.7680	0.2409	0.046*	
O1	0.6875 (3)	0.9631 (2)	0.47878 (17)	0.0872 (7)	
N2	0.4265 (2)	0.49916 (19)	0.39447 (16)	0.0465 (5)	
H2A	0.3987	0.4100	0.4353	0.056*	
O2	0.7270 (2)	0.77039 (17)	0.43616 (14)	0.0554 (5)	
C2	0.6977 (2)	0.9558 (2)	0.2084 (2)	0.0386 (5)	
O3	0.8103 (2)	0.52264 (18)	0.29747 (17)	0.0636 (5)	
C3	0.6956 (2)	1.0904 (2)	0.1197 (2)	0.0395 (6)	
O4	0.6464 (2)	0.32942 (17)	0.38907 (16)	0.0633 (5)	
C4	0.7094 (3)	1.1362 (2)	0.0044 (2)	0.0479 (6)	
H4A	0.7174	1.0699	−0.0292	0.058*	
F	0.8690 (2)	0.7816 (2)	−0.22102 (14)	0.0854 (6)	
C5	0.7110 (3)	1.2785 (3)	−0.0579 (2)	0.0559 (7)	
H5B	0.7218	1.3084	−0.1342	0.067*	
C6	0.6967 (3)	1.3798 (3)	−0.0089 (3)	0.0627 (8)	
H6A	0.6972	1.4757	−0.0534	0.075*	
C7	0.6819 (3)	1.3414 (3)	0.1023 (3)	0.0587 (7)	
H7A	0.6717	1.4090	0.1348	0.070*	
C8	0.6827 (3)	1.1961 (2)	0.1658 (2)	0.0464 (6)	
C9	0.6911 (3)	0.9871 (2)	0.3010 (2)	0.0443 (6)	
C10	0.7028 (3)	0.8940 (3)	0.4101 (2)	0.0485 (6)	
C11	0.7075 (5)	0.8835 (4)	0.5877 (3)	0.1118 (15)	
H11A	0.6940	0.9428	0.6304	0.168*	
H11B	0.8033	0.8555	0.5773	0.168*	
H11C	0.6386	0.7997	0.6272	0.168*	
C12	0.5725 (3)	0.7045 (2)	0.28357 (19)	0.0384 (5)	
C13	0.4254 (3)	0.7349 (2)	0.30284 (19)	0.0408 (6)	
C14	0.3562 (3)	0.8584 (3)	0.2647 (2)	0.0502 (7)	
H14A	0.4102	0.9463	0.2179	0.060*	
C15	0.2095 (3)	0.8478 (3)	0.2971 (2)	0.0616 (8)	
H15A	0.1641	0.9295	0.2720	0.074*	
C16	0.1249 (3)	0.7167 (3)	0.3675 (3)	0.0649 (8)	
H16A	0.0250	0.7130	0.3880	0.078*	
C17	0.1881 (3)	0.5942 (3)	0.4064 (2)	0.0587 (7)	
H17A	0.1327	0.5073	0.4538	0.070*	
C18	0.3376 (3)	0.6042 (2)	0.37256 (19)	0.0441 (6)	
C19	0.5672 (3)	0.5583 (2)	0.34119 (19)	0.0399 (6)	
C20	0.6760 (3)	0.4588 (3)	0.3467 (2)	0.0460 (6)	
C21	0.9232 (3)	0.4299 (3)	0.2909 (3)	0.0868 (11)	
H21A	1.0152	0.4869	0.2540	0.130*	
H21B	0.9091	0.3751	0.2490	0.130*	
H21C	0.9203	0.3662	0.3653	0.130*	
C22	0.7510 (2)	0.8014 (2)	0.09177 (19)	0.0386 (6)	
C23	0.6484 (3)	0.7791 (3)	0.0436 (2)	0.0495 (6)	
H23A	0.5512	0.7679	0.0834	0.059*	
C24	0.6868 (3)	0.7731 (3)	−0.0611 (2)	0.0527 (7)	
H24A	0.6170	0.7588	−0.0924	0.063*	
C25	0.8294 (3)	0.7885 (3)	−0.1176 (2)	0.0543 (7)	
C26	0.9337 (3)	0.8114 (3)	−0.0755 (3)	0.0698 (9)	
H26A	1.0304	0.8225	−0.1164	0.084*	
C27	0.8938 (3)	0.8180 (3)	0.0304 (2)	0.0581 (7)	
H27A	0.9648	0.8339	0.0600	0.070*	

### Atomic displacement parameters (Å^2^)

**Table:**

	*U*^11^	*U*^22^	*U*^33^	*U*^12^	*U*^13^	*U*^23^
N1	0.0702 (15)	0.0321 (11)	0.0561 (14)	0.0069 (10)	−0.0203 (11)	−0.0209 (10)
C1	0.0363 (13)	0.0298 (12)	0.0485 (14)	0.0036 (10)	−0.0142 (11)	−0.0142 (10)
O1	0.158 (2)	0.0616 (13)	0.0675 (14)	0.0279 (14)	−0.0540 (15)	−0.0379 (12)
N2	0.0553 (13)	0.0267 (10)	0.0480 (12)	−0.0034 (9)	−0.0092 (10)	−0.0086 (9)
O2	0.0728 (13)	0.0346 (10)	0.0542 (11)	0.0024 (8)	−0.0202 (9)	−0.0113 (8)
C2	0.0337 (12)	0.0299 (12)	0.0494 (14)	0.0010 (9)	−0.0114 (11)	−0.0131 (11)
O3	0.0548 (12)	0.0421 (10)	0.0936 (15)	0.0147 (9)	−0.0217 (11)	−0.0262 (10)
C3	0.0353 (13)	0.0292 (12)	0.0511 (15)	0.0006 (10)	−0.0136 (11)	−0.0119 (11)
O4	0.0838 (14)	0.0297 (10)	0.0768 (13)	0.0119 (9)	−0.0245 (11)	−0.0209 (9)
C4	0.0477 (15)	0.0340 (13)	0.0594 (17)	−0.0002 (11)	−0.0175 (13)	−0.0139 (12)
F	0.0945 (14)	0.1087 (15)	0.0628 (11)	0.0344 (11)	−0.0181 (10)	−0.0482 (11)
C5	0.0586 (17)	0.0418 (15)	0.0601 (17)	−0.0014 (12)	−0.0234 (14)	−0.0074 (13)
C6	0.071 (2)	0.0319 (14)	0.078 (2)	0.0014 (13)	−0.0318 (17)	−0.0065 (14)
C7	0.076 (2)	0.0311 (13)	0.074 (2)	0.0059 (13)	−0.0321 (16)	−0.0184 (13)
C8	0.0462 (15)	0.0326 (13)	0.0599 (17)	0.0033 (11)	−0.0173 (12)	−0.0165 (12)
C9	0.0514 (15)	0.0285 (12)	0.0521 (15)	0.0017 (10)	−0.0146 (12)	−0.0152 (11)
C10	0.0569 (17)	0.0396 (14)	0.0511 (16)	−0.0015 (12)	−0.0172 (13)	−0.0190 (12)
C11	0.192 (5)	0.096 (3)	0.071 (2)	0.030 (3)	−0.064 (3)	−0.041 (2)
C12	0.0473 (14)	0.0292 (12)	0.0387 (13)	0.0027 (10)	−0.0115 (11)	−0.0142 (10)
C13	0.0462 (14)	0.0312 (12)	0.0402 (13)	−0.0007 (10)	−0.0068 (11)	−0.0128 (10)
C14	0.0493 (16)	0.0332 (13)	0.0606 (17)	0.0054 (11)	−0.0125 (13)	−0.0128 (12)
C15	0.0484 (17)	0.0472 (16)	0.080 (2)	0.0104 (13)	−0.0138 (15)	−0.0185 (15)
C16	0.0446 (16)	0.0591 (18)	0.075 (2)	0.0039 (13)	−0.0047 (14)	−0.0182 (16)
C17	0.0469 (17)	0.0479 (16)	0.0610 (18)	−0.0098 (13)	−0.0009 (13)	−0.0098 (13)
C18	0.0522 (16)	0.0342 (13)	0.0399 (14)	−0.0009 (11)	−0.0080 (12)	−0.0122 (11)
C19	0.0498 (15)	0.0288 (12)	0.0415 (13)	0.0022 (10)	−0.0135 (11)	−0.0141 (10)
C20	0.0596 (17)	0.0342 (13)	0.0483 (15)	0.0067 (12)	−0.0182 (13)	−0.0190 (11)
C21	0.066 (2)	0.073 (2)	0.130 (3)	0.0350 (18)	−0.033 (2)	−0.047 (2)
C22	0.0382 (13)	0.0279 (11)	0.0464 (14)	0.0021 (9)	−0.0085 (11)	−0.0137 (10)
C23	0.0410 (15)	0.0523 (15)	0.0546 (16)	0.0014 (12)	−0.0085 (12)	−0.0240 (13)
C24	0.0574 (18)	0.0510 (16)	0.0564 (17)	0.0068 (13)	−0.0199 (14)	−0.0259 (13)
C25	0.0631 (19)	0.0544 (16)	0.0486 (16)	0.0144 (13)	−0.0121 (14)	−0.0270 (13)
C26	0.0444 (17)	0.094 (2)	0.072 (2)	0.0155 (16)	−0.0050 (15)	−0.0420 (19)
C27	0.0397 (15)	0.0742 (19)	0.0661 (19)	0.0057 (13)	−0.0142 (14)	−0.0345 (16)

### Geometric parameters (Å, º)

**Table:**

N1—C8	1.364 (3)	C11—H11A	0.9600
N1—C9	1.386 (3)	C11—H11B	0.9600
N1—H1A	0.8600	C11—H11C	0.9600
C1—C12	1.513 (3)	C12—C19	1.385 (3)
C1—C2	1.519 (3)	C12—C13	1.434 (3)
C1—C22	1.522 (3)	C13—C14	1.410 (3)
C1—H1B	0.9800	C13—C18	1.413 (3)
O1—C10	1.328 (3)	C14—C15	1.362 (4)
O1—C11	1.453 (4)	C14—H14A	0.9300
N2—C18	1.366 (3)	C15—C16	1.403 (4)
N2—C19	1.372 (3)	C15—H15A	0.9300
N2—H2A	0.8600	C16—C17	1.371 (4)
O2—C10	1.209 (3)	C16—H16A	0.9300
C2—C9	1.379 (3)	C17—C18	1.389 (4)
C2—C3	1.443 (3)	C17—H17A	0.9300
O3—C20	1.329 (3)	C19—C20	1.456 (3)
O3—C21	1.446 (3)	C21—H21A	0.9600
C3—C4	1.409 (3)	C21—H21B	0.9600
C3—C8	1.411 (3)	C21—H21C	0.9600
O4—C20	1.216 (3)	C22—C27	1.376 (3)
C4—C5	1.368 (3)	C22—C23	1.393 (3)
C4—H4A	0.9300	C23—C24	1.375 (3)
F—C25	1.363 (3)	C23—H23A	0.9300
C5—C6	1.398 (4)	C24—C25	1.359 (4)
C5—H5B	0.9300	C24—H24A	0.9300
C6—C7	1.362 (4)	C25—C26	1.351 (4)
C6—H6A	0.9300	C26—C27	1.393 (4)
C7—C8	1.400 (3)	C26—H26A	0.9300
C7—H7A	0.9300	C27—H27A	0.9300
C9—C10	1.454 (3)		
			
C8—N1—C9	108.9 (2)	C13—C12—C1	128.2 (2)
C8—N1—H1A	125.6	C14—C13—C18	117.7 (2)
C9—N1—H1A	125.6	C14—C13—C12	134.8 (2)
C12—C1—C2	113.09 (19)	C18—C13—C12	107.4 (2)
C12—C1—C22	110.81 (18)	C15—C14—C13	119.3 (2)
C2—C1—C22	114.33 (18)	C15—C14—H14A	120.3
C12—C1—H1B	106.0	C13—C14—H14A	120.3
C2—C1—H1B	106.0	C14—C15—C16	121.7 (3)
C22—C1—H1B	106.0	C14—C15—H15A	119.1
C10—O1—C11	116.2 (2)	C16—C15—H15A	119.1
C18—N2—C19	109.29 (19)	C17—C16—C15	120.7 (3)
C18—N2—H2A	125.4	C17—C16—H16A	119.6
C19—N2—H2A	125.4	C15—C16—H16A	119.6
C9—C2—C3	105.93 (19)	C16—C17—C18	117.8 (2)
C9—C2—C1	123.5 (2)	C16—C17—H17A	121.1
C3—C2—C1	130.5 (2)	C18—C17—H17A	121.1
C20—O3—C21	116.5 (2)	N2—C18—C17	129.7 (2)
C4—C3—C8	117.4 (2)	N2—C18—C13	107.6 (2)
C4—C3—C2	135.5 (2)	C17—C18—C13	122.7 (2)
C8—C3—C2	107.1 (2)	N2—C19—C12	109.9 (2)
C5—C4—C3	119.7 (2)	N2—C19—C20	116.4 (2)
C5—C4—H4A	120.2	C12—C19—C20	133.4 (2)
C3—C4—H4A	120.2	O4—C20—O3	123.3 (2)
C4—C5—C6	121.3 (3)	O4—C20—C19	123.0 (2)
C4—C5—H5B	119.4	O3—C20—C19	113.7 (2)
C6—C5—H5B	119.4	O3—C21—H21A	109.5
C7—C6—C5	121.5 (2)	O3—C21—H21B	109.5
C7—C6—H6A	119.2	H21A—C21—H21B	109.5
C5—C6—H6A	119.2	O3—C21—H21C	109.5
C6—C7—C8	117.3 (3)	H21A—C21—H21C	109.5
C6—C7—H7A	121.4	H21B—C21—H21C	109.5
C8—C7—H7A	121.4	C27—C22—C23	117.5 (2)
N1—C8—C7	128.8 (2)	C27—C22—C1	121.2 (2)
N1—C8—C3	108.2 (2)	C23—C22—C1	121.4 (2)
C7—C8—C3	122.9 (2)	C24—C23—C22	121.9 (2)
C2—C9—N1	109.8 (2)	C24—C23—H23A	119.0
C2—C9—C10	129.3 (2)	C22—C23—H23A	119.0
N1—C9—C10	120.8 (2)	C25—C24—C23	118.2 (3)
O2—C10—O1	123.1 (2)	C25—C24—H24A	120.9
O2—C10—C9	125.4 (2)	C23—C24—H24A	120.9
O1—C10—C9	111.4 (2)	C26—C25—C24	122.6 (3)
O1—C11—H11A	109.5	C26—C25—F	118.6 (3)
O1—C11—H11B	109.5	C24—C25—F	118.8 (3)
H11A—C11—H11B	109.5	C25—C26—C27	118.8 (3)
O1—C11—H11C	109.5	C25—C26—H26A	120.6
H11A—C11—H11C	109.5	C27—C26—H26A	120.6
H11B—C11—H11C	109.5	C22—C27—C26	121.1 (3)
C19—C12—C13	105.8 (2)	C22—C27—H27A	119.5
C19—C12—C1	125.9 (2)	C26—C27—H27A	119.5
			
C12—C1—C2—C9	66.6 (3)	C1—C12—C13—C18	175.5 (2)
C22—C1—C2—C9	−165.3 (2)	C18—C13—C14—C15	0.8 (4)
C12—C1—C2—C3	−114.3 (3)	C12—C13—C14—C15	177.2 (3)
C22—C1—C2—C3	13.8 (3)	C13—C14—C15—C16	−0.1 (4)
C9—C2—C3—C4	174.6 (3)	C14—C15—C16—C17	0.1 (5)
C1—C2—C3—C4	−4.6 (4)	C15—C16—C17—C18	−0.8 (4)
C9—C2—C3—C8	−2.0 (3)	C19—N2—C18—C17	178.4 (3)
C1—C2—C3—C8	178.8 (2)	C19—N2—C18—C13	−0.1 (3)
C8—C3—C4—C5	0.5 (3)	C16—C17—C18—N2	−176.8 (3)
C2—C3—C4—C5	−175.8 (3)	C16—C17—C18—C13	1.6 (4)
C3—C4—C5—C6	−1.1 (4)	C14—C13—C18—N2	177.1 (2)
C4—C5—C6—C7	0.6 (4)	C12—C13—C18—N2	−0.2 (3)
C5—C6—C7—C8	0.5 (4)	C14—C13—C18—C17	−1.6 (4)
C9—N1—C8—C7	−176.3 (3)	C12—C13—C18—C17	−178.8 (2)
C9—N1—C8—C3	0.1 (3)	C18—N2—C19—C12	0.3 (3)
C6—C7—C8—N1	174.9 (3)	C18—N2—C19—C20	−174.2 (2)
C6—C7—C8—C3	−1.0 (4)	C13—C12—C19—N2	−0.4 (3)
C4—C3—C8—N1	−176.1 (2)	C1—C12—C19—N2	−175.7 (2)
C2—C3—C8—N1	1.2 (3)	C13—C12—C19—C20	172.8 (2)
C4—C3—C8—C7	0.6 (4)	C1—C12—C19—C20	−2.5 (4)
C2—C3—C8—C7	177.9 (2)	C21—O3—C20—O4	3.2 (4)
C3—C2—C9—N1	2.1 (3)	C21—O3—C20—C19	−174.6 (2)
C1—C2—C9—N1	−178.7 (2)	N2—C19—C20—O4	4.8 (4)
C3—C2—C9—C10	−173.9 (2)	C12—C19—C20—O4	−168.0 (3)
C1—C2—C9—C10	5.3 (4)	N2—C19—C20—O3	−177.5 (2)
C8—N1—C9—C2	−1.4 (3)	C12—C19—C20—O3	9.7 (4)
C8—N1—C9—C10	175.0 (2)	C12—C1—C22—C27	−145.7 (2)
C11—O1—C10—O2	2.5 (4)	C2—C1—C22—C27	85.1 (3)
C11—O1—C10—C9	−175.7 (3)	C12—C1—C22—C23	33.8 (3)
C2—C9—C10—O2	3.3 (4)	C2—C1—C22—C23	−95.4 (3)
N1—C9—C10—O2	−172.3 (2)	C27—C22—C23—C24	0.2 (4)
C2—C9—C10—O1	−178.5 (2)	C1—C22—C23—C24	−179.3 (2)
N1—C9—C10—O1	5.9 (3)	C22—C23—C24—C25	0.5 (4)
C2—C1—C12—C19	−148.6 (2)	C23—C24—C25—C26	−1.0 (4)
C22—C1—C12—C19	81.6 (3)	C23—C24—C25—F	179.4 (2)
C2—C1—C12—C13	37.2 (3)	C24—C25—C26—C27	0.6 (5)
C22—C1—C12—C13	−92.7 (3)	F—C25—C26—C27	−179.8 (2)
C19—C12—C13—C14	−176.2 (3)	C23—C22—C27—C26	−0.6 (4)
C1—C12—C13—C14	−1.1 (4)	C1—C22—C27—C26	178.9 (2)
C19—C12—C13—C18	0.4 (2)	C25—C26—C27—C22	0.1 (5)

### Hydrogen-bond geometry (Å, º)

Cg1, Cg4 and Cg5 are the centroids of the N1-ring, C14-ring and C22-ring.

**Table:**

*D*—H···*A*	*D*—H	H···*A*	*D*···*A*	*D*—H···*A*
N1—H1*A*···O4^i^	0.86	2.07	2.903 (3)	162
N2—H2*A*···O2^ii^	0.86	2.07	2.892 (3)	159
C4—H4*A*···*Cg*5	0.93	2.82	3.622 (3)	146
C5—H5*B*···*Cg*4^iii^	0.93	2.84	3.705 (3)	156
C26—H26*A*···*Cg*1^iv^	0.93	2.69	3.587 (4)	161

Symmetry codes: (i) *x*, *y*+1, *z*; (ii) −*x*+1, −*y*+1, −*z*+1; (iii) −*x*+1, −*y*+2, −*z*; (iv) −*x*+2, −*y*+2, −*z*.
